# An optimised method for intact nuclei isolation from diatoms

**DOI:** 10.1038/s41598-021-81238-z

**Published:** 2021-01-18

**Authors:** Rossella Annunziata, Cecilia Balestra, Pina Marotta, Antonella Ruggiero, Francesco Manfellotto, Giovanna Benvenuto, Elio Biffali, Maria Immacolata Ferrante

**Affiliations:** Stazione Zoologica Anton Dohrn, 80121 Napoli, Italy

**Keywords:** Marine biology, Functional genomics

## Abstract

Due to their abundance in the oceans, their extraordinary biodiversity and the increasing use for biotech applications, the study of diatom biology is receiving more and more attention in the recent years. One of the limitations in developing molecular tools for diatoms lies in the peculiar nature of their cell wall, that is made of silica and organic molecules and that hinders the application of standard methods for cell lysis required, for example, to extract organelles. In this study we present a protocol for intact nuclei isolation from diatoms that was successfully applied to three different species: two pennates, *Pseudo-nitzschia multistriata* and *Phaeodactylum tricornutum,* and one centric diatom species, *Chaetoceros diadema*. Intact nuclei were extracted by treatment with acidified NH_4_F solution combined to low intensity sonication pulses and separated from cell debris via FAC-sorting upon incubation with SYBR Green. Microscopy observations confirmed the integrity of isolated nuclei and high sensitivity DNA electrophoresis showed that genomic DNA extracted from isolated nuclei has low degree of fragmentation. This protocol has proved to be a flexible and versatile method to obtain intact nuclei preparations from different diatom species and it has the potential to speed up applications such as epigenetic explorations as well as single cell (“single nuclei”) genomics, transcriptomics and proteomics in different diatom species.

## Introduction

Diatoms are unicellular eukaryotic microalgae and constitute a major group in the phytoplankton community^1^. They are distributed worldwide inhabiting marine and freshwater aquatic ecosystems^2^ as well as terrestrial ecosystems and contribute to more than 20% of the global primary production, as much as all terrestrial rainforests together^3^. These microalgae are also being investigated for the production of bioproducts with pharmaceutical and nutraceutical applications as well as for the production of biofuel and nanomaterials^4,5^.

Diatoms can be divided into three groups based on the ultrastructure of their cell wall: Coscinodiscophyceae (radial centrics), which show radial symmetry; Mediophyceae (including Thalassiosirales), which exhibit polar symmetry, and Bacillariophyceae (pennates), which show bilateral symmetry^6–8^. In the last decades, several centric and pennate diatom genomes have been released^9–14^ offering a unique opportunity to investigate diatom genomic organization and function. The huge gain of knowledge brought by diatom sequenced genomes has been complemented by the development of advanced molecular tools such as stable genetic transformation through biolistic bombardment^15^ and genome editing techniques^16,17^.

The progress in the development of molecular tools coupled to the release of genome and transcriptome sequences revealed sophisticated mechanisms regulating diatom biology in response to environmental cues. Examples are the expanded number of cyclins used to finely regulate cell cycle^18,19^, the selective use of a wide range of photoreceptors mediating responses to different light wavelengths^20^, and the tight regulation of diurnal cellular activities through transcriptional timekeepers^21^.

Diatoms can exist as single cells or form chains and their size can range from few micrometers to few millimeters^7^. Their cell wall, the frustule, consists of inorganic silica and organic macromolecules such as polysaccharides, long-chain polyamines and proteins^22^. To date, four families of diatom cell wall proteins have been identified: frustulins, pleuralins, silaffins and the p150 family^11,23^.

Frustules present species-specific diversified morphologies and chemical compositions^24,25^ and are composed of two valves of unequal size, overlapping as a lid with its box. During their vegetative cycle, diatoms divide mitotically and at each round of division the hypotheca will become the big valve of one of the two daughter cells, causing progressive cell size reduction of the population. To escape miniaturization and restore initial cell size, diatoms can reproduce sexually but only when cells reach a definite size threshold^26^. For example, cells of the heterothallic *Pseudo-nitzschia multistriata*, whose size can range from 80 to around 20 µm, become competent for sexual reproduction when their size is below 55 µm. Recent works are showing sexual reproduction to be a finely regulated process in diatoms^14,27–29^.

The peculiar composition of diatom cells wall often represents an obstacle for the direct application of molecular techniques developed for other organisms, one example being the isolation of cellular organelles. Cell fractionation has been used to isolate thylakoid membranes from a few species such as *Phaeodactylum tricornutum*^30^, *Cylindrotheca fusiformis*^31^, *Chaetoceros gracilis*^32^ and *Cyclotella meneghiniana*^33^, while intact plastids have been isolated from *Odontella sinensis* and *Coscinodiscus granii*^34^ and more recently from *Thalassiosira pseudonana* cells from which, for the first time, also mitochondria have been extracted^35^. While examples of chloroplast and mitochondria isolations are accessible, and despite the existence of few publications reporting the application in diatoms of methods requiring nuclei isolation such as MNase digestion, Chromatin immune-precipitation (Chip-seq) and histone extraction^36–38^, to date detailed, step-by-step protocols for intact nuclei isolation from diatom cells are not available.

In this study we present a rapid and flexible protocol for intact nuclei isolation that has been setup for the pennate *P. multistriata* and adapted to one centric (*Chaetoceros diadema*) and one additional pennate (*P. tricornutum*) diatom species. This methodology has the potential to speedup research in areas that are still little explored in diatoms, such as the regulation of chromatin accessibility by application of ATAC-Seq (Assay for Transposase-Accessible Chromatin using Sequencing)^39^. This agile nuclei isolation protocol could also prompt the study of cell-to-cell variability on a genomic scale, through gDNA- and RNA-Seq on single nuclei extracted from cells^40,41^.

## Results

### Cell disruption for nuclei isolation

The protocol presented in this section has been developed to extract intact nuclei from *P. multistriata* cells and then adapted to two other diatom species. It consists of a combination of acidified NH_4_F solution treatments and short sonication pulses followed by FAC-sorting. To be sure that nuclei extraction was performed in an appropriate chemical environment, sonication of cell cultures and all the subsequent steps were performed in a low ionic strength Nuclei Isolation Buffer (NIB, 10 mM Tris–HCl pH 7.4; 10 mM NaCl; 3 mM MgCl_2_) supplemented with 0.1% IGEPAL. Before switching to sonication, numerous attempts to break *P. multistriata* cells combining different incubation times in NH_4_F solution and glass beads treatments have been made, but results were not satisfying (Table S1).

**Figure 1:**
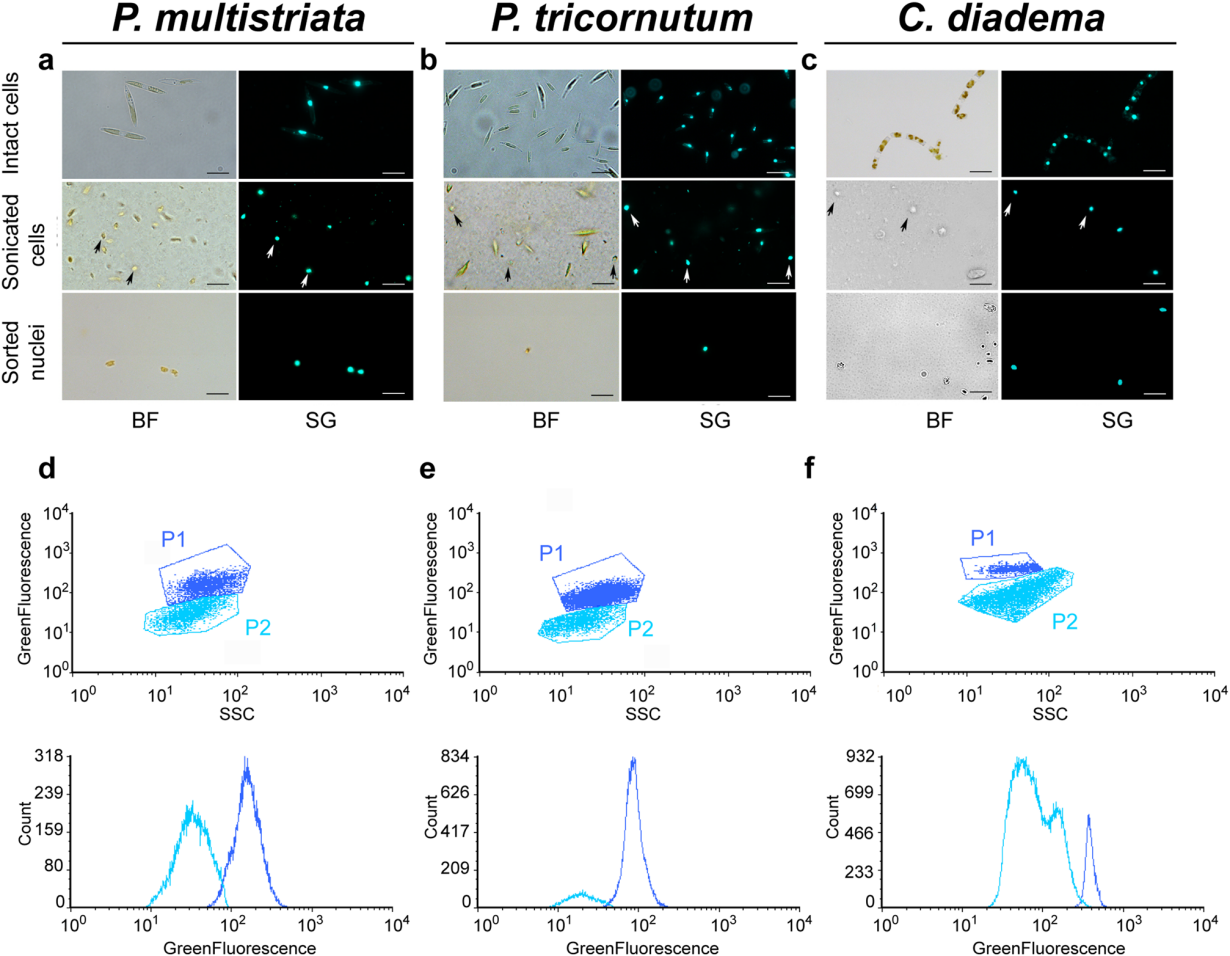
Isolation of intact nuclei from *Pseudo-nitzschia multistriata*, *Phaeodactylum tricornutum*, and *Chaetoceros diadema*. Bright-field and fluorescent microscopy images of intact cells, sonicated cells and sorted nuclei for *P. multistriata* (**a**), *P. tricornutum* (**b**) and *C. diadema* (**c**), scale bar 5 μm. Dot plots (up) and histograms (down) of flow cytometry analysis for nuclei isolation in *P. multistriata* (**d**), *P. tricornutum* (**e**) and *C. diadema* (**f**). In dark blue P1 indicates the gate containing extracted nuclei; in light blue P2 indicates the gate containing cellular residues. White and black arrows point to extracted nuclei. BF (Bright Field), SG (SYBR Green I), SSC (Side-Scatter). Graphs in (**d**), (**e**) and (**f**) were drawn with FCS Express 6 Flow v 6.06.0025, DeNovo Software, USA.

Similar tests were then performed using sonication instead of glass beads treatments (Table S2), aiming to find the optimal setup enabling cell disruption and preserving nuclei quality. The disruption of cells and the presence of intact nuclei in the sonicated samples were tested by careful observations under fluorescence light, upon incubation with SYBR Green I, which stains DNA (Fig. 1a). When broken cells represented at least 60–70% of the observed cells, no more sonication pulses were applied and the sample was considered suitable for the subsequent steps of the protocol. The optimal setup to extract nuclei from *P. multistriata* cells consisted in 10′ incubation in NH_4_F solution followed by 5–12 sonication pulses (depending on the tested strain) at 20% intensity (corresponding to 40 W). We observed that *P. multistriata* strains with a greater average cell size (70 µm to 50 µm) could be broken more easily compared to smaller cells (50 µm to 20 µm).

In order to verify if the procedure could be applied to other diatom species, the protocol was tested on one more pennate diatom (*P. tricornutum*) (Fig. 1b) and one centric diatom species (*C. diadema*) (Fig. 1c). For these species, the duration of the NH_4_F treatment was not modified, while the number of sonication pulses had to be adapted. In particular, 16–20 pulses were needed for *P. tricornutum* cells and 6–7 pulses for *C. diadema*. The protocol resulted highly reproducible for both *P. multistriata* and *C. diadema*, while some variability in the number of sonication pulses needed to break the cells was observed for *P. tricornutum*. Nevertheless, it was possible to isolate nuclei from all the tested species although they sometimes presented cell wall residues, especially for *P. tricornutum*. For *P. multistriata* the experiment was reproduced at least 40 times, for the other two species the protocol was successfully replicated and intact nuclei were visualized at least 2 times. At the end of each experiment 50–100 nuclei were observed under fluorescence light to check the efficiency of nuclei extraction and their integrity.

### Separation of nuclei from cell debris

Once cells have been sonicated and nuclei extraction achieved, the resulting sample is composed of unbroken cells, extracted nuclei, cell residues and, in some cases, bacteria (see sonicated cells in Fig. 1a–c). Density gradient centrifugation methodologies were tested to separate nuclei from the other components (e.g.: Percoll gradient separation and sucrose gradient centrifugation) but none of the attempts allowed an efficient separation of nuclei from cell debris (and bacteria, when present). We thus decided to subject the mixture to flow cytometry and sort nuclei upon incubation with SYBR Green I. The combination of Side-scatter (SSC) and Green Fluorescence (530/40 nm) was used to discriminate the nuclei population and sort them (Fig. 1d–f). Sorting purity was verified by fluorescence microscopy (sorted nuclei in Fig. 1a–c). Sorting procedure was performed using the ‘1 drop pure’ sorting mode, ensuring the absence of non-target particles within the target cell drop and the drops immediately surrounding the cell. This procedure allowed the isolation of pure nuclei preparations in a relatively short time: around 1 million nuclei were isolated in 90 min of sorting procedure.

### Microscopic analyses of isolated nuclei

**Figure 2:**
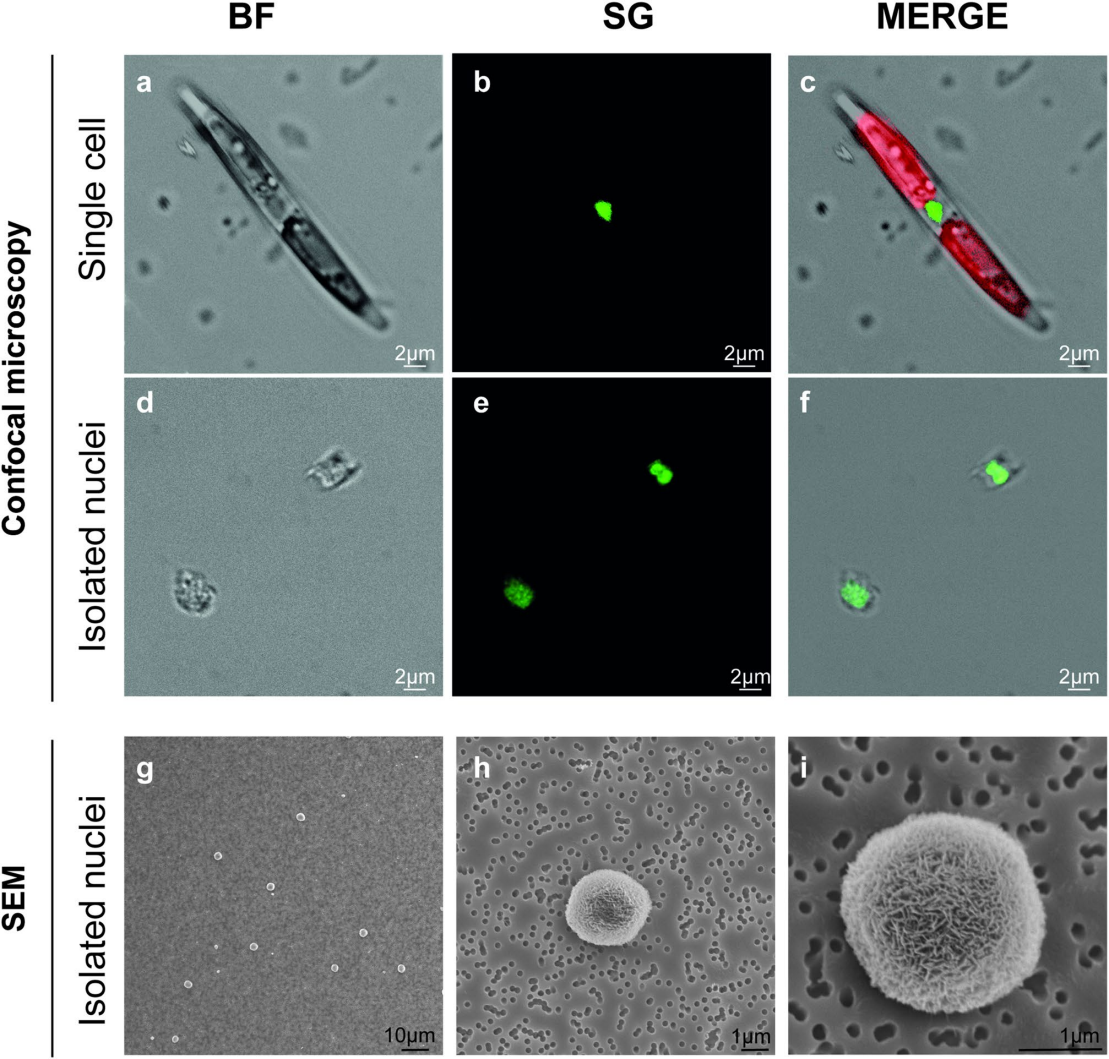
Microscopy analysis of *P. multistriata* cell and isolated nuclei. Confocal (**a**–**f**) and SEM (**g**–**i**) images of *P. multistriata* isolated cell (**a**–**c**) and nuclei (**d**–**i**) after the FAC-sorting procedure. In C chlorophyll autofluorescence is visible in red. BF (bright field), SG (SYBR Green I).

**Figure 3:**
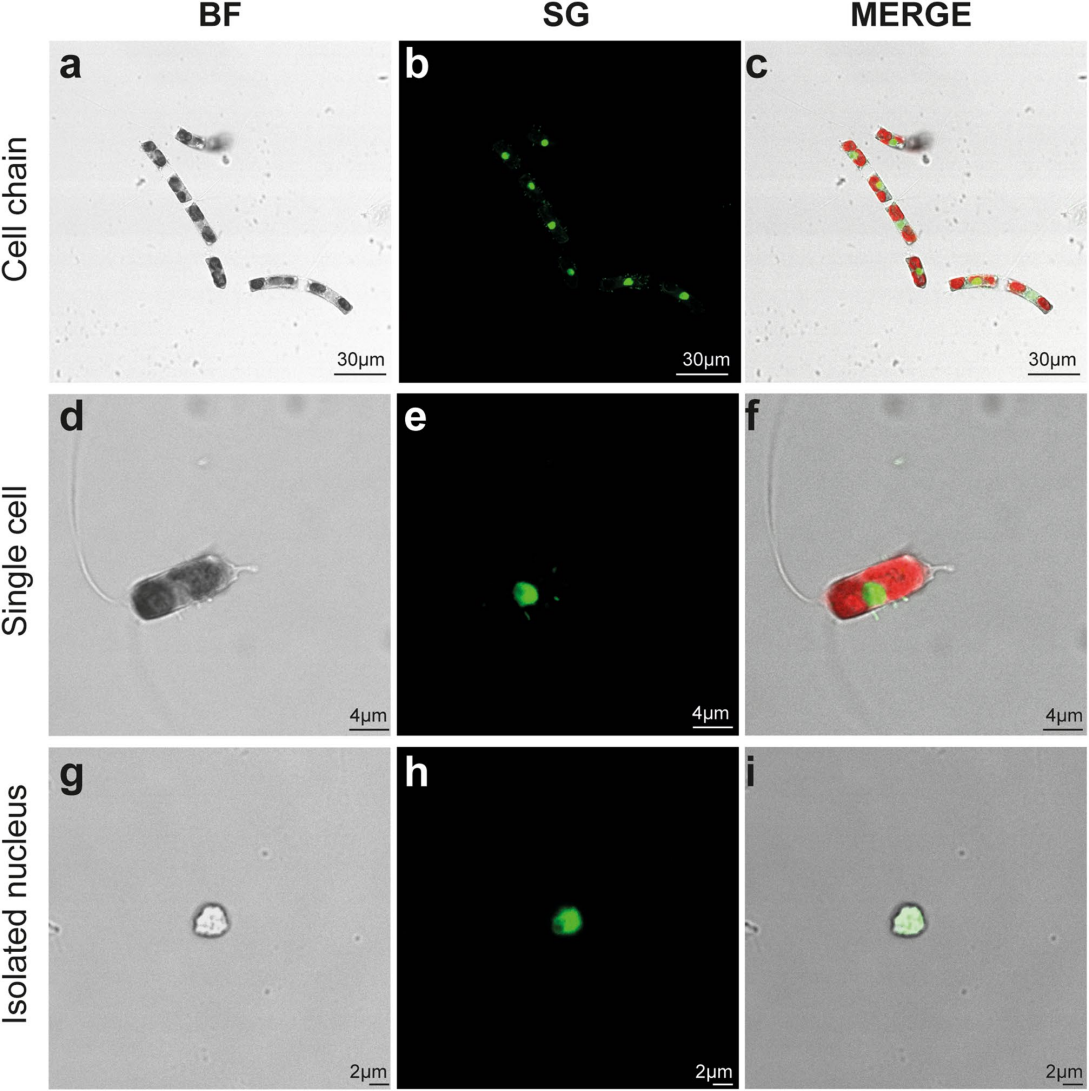
Confocal microscopy analysis of *C. diadema* cells and isolated nuclei. Bright-field and fluorescence microscopy images of intact cell chains (**a**–**c**), single cell (**d**–**f**) and sorted nuclei (**g**–**i**) for *C. diadema*. In (**c**) and (**f**) chlorophyll autofluorescence is visible in red. BF (Bright Field), SG (SYBR Green I).

To check if the entire experimental procedure had an effect on nuclei integrity, confocal observations were performed for *P. multistriata* (Fig. 2a–f) and *C. diadema* (Fig. 3) cells and nuclei. Comparative confocal microscopy on both intact cells (Fig. 2a–c) and isolated nuclei (d–f) revealed that *P. multistriata* isolated nuclei showed an overall intact structure although in some cases they presented attached cell wall residues. *P. multistriata* nuclei integrity was tested with confocal microscopy and confirmed by SEM acquisitions (Fig. 2g–i) in which at least 100 nuclei were imaged and resulted free of cell wall residues, probably as a consequence of the fixation and washing steps required for the preparation of the samples before microscopy observation (see Materials & Methods section). Confocal observations on *C. diadema* cells (Fig. 3a–f) and isolated nuclei (Fig. 3g–i) also confirmed that nuclei structure was not affected by the procedure (at least 40 nuclei were imaged to confirm their integrity).

To specifically prove that the FAC-sorting procedure was not altering nuclei morphology, preparations of *P. multistriata* nuclei before and after FAC-sorting were observed at SEM (Supplementary Fig. 1). Although the nuclei envelopes were not clearly visible (likely due to the presence of precipitated residues of fixative), nuclei shape before (Supplementary Fig. 1 a, c) and after the FAC-sorting procedure (Supplementary Fig. 1 b, d) appeared unaltered, demonstrating the suitability of this methodology to the protocol. Moreover, the SEM images clearly showed the presence of abundant cellular debris in the unsorted samples that was instead absent from the FAC-sorted nuclei preparations.

### Analyses of genomic DNA extracted from isolated nuclei and whole cells

**Figure 4:**
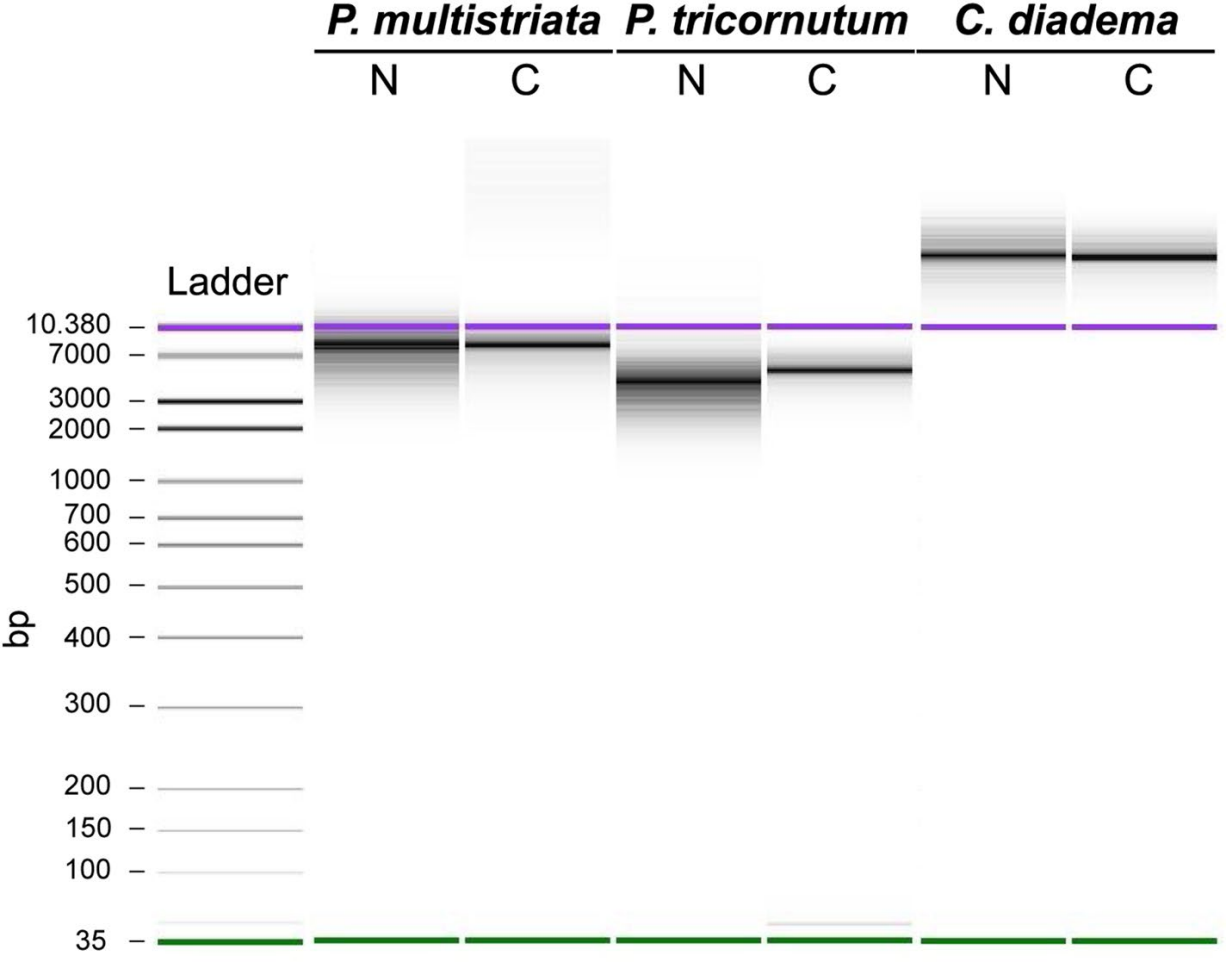
Quality control of genomic DNA extracted from *P. multistriata*, *P. tricornutum*, and *C. diadema*. High sensitivity DNA electrophoresis (Agilent 2100 Bioanalyzer System) of gDNAs extracted from intact cells and isolated nuclei from *P. multistriata*, *P. tricornutum* and *C. diadema*. N (nuclei), C (cells), bp (base pairs). In purple and green the higher and the lower bands of the DNA-ladder used as reference.

To explore potential applications of the developed experimental procedure, we tested the possibility to extract gDNA from intact nuclei. Extractions were performed in parallel from isolated nuclei and intact cells for all diatom species (12–40 ng of gDNA were extracted starting from 500.000–1.000.000 nuclei while 200–700 ng were extracted starting from 500.000–3.000.000 cells) and gDNAs were analyzed through high sensitivity DNA electrophoresis (Fig. 4). The gDNA profiles of samples extracted from isolated nuclei for *P. multistriata*, *P. tricornutum* and *C. diadema* did not show fragmentation at least at low molecular weights, similarly to those extracted from whole cells (Fig. 4) suggesting that the applied experimental procedure allows to obtain gDNA albeit the degrees of integrity at high molecular weights remain to be assessed.

### Step by step protocol for intact nuclei isolation from diatom cells

**Figure 5:**
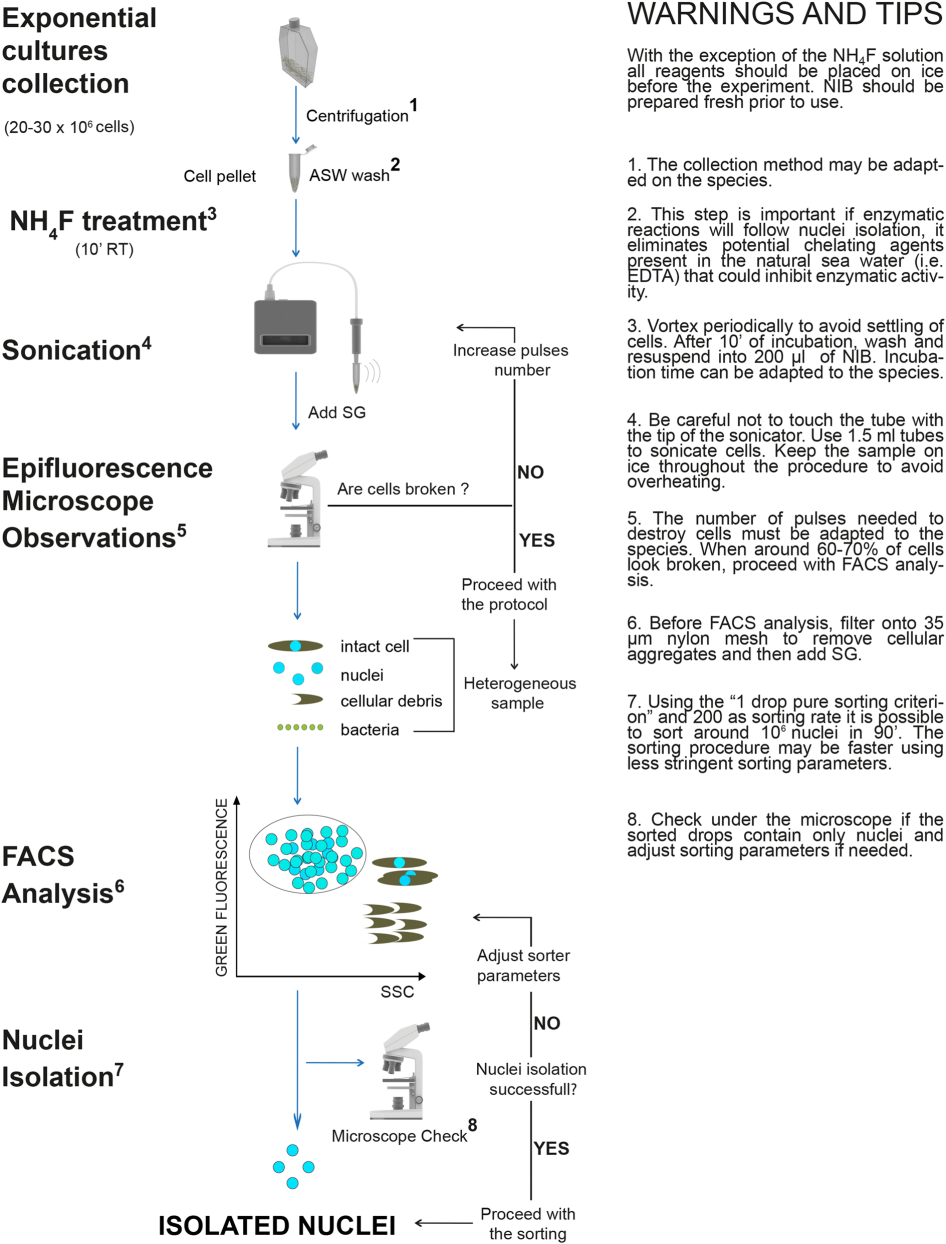
Intact nuclei isolation from diatom cells: the experimental workflow. Step by step protocol and useful warnings and tips to isolate intact nuclei from diatom cells. Main steps are indicated on the left: diatoms cells are harvested via centrifugation; NH_4_F treatment is performed to weaken diatom cell wall; sonication is applied to destroy frustules allowing nuclei extraction which is verified using epifluorescence microscopy; nuclei are sorted by FACS from a mixed sample containing also cellular debris and, in some cases, bacteria, obtaining a clean preparation of isolated nuclei. ASW (Artificial Sea Water), RT (Room Temperature), SG (SYBR Green I), NIB (Nuclei Isolation Buffer), FACS (Fluorescence Activated Cell Sorting), SSC (Side-Scatter). Images used for the figure were adapted from “Library of Science and Medical Illustrations CC BY-NC-SA 4.0”.

All the tests described above allowed us to design a protocol for nuclei isolation from *P. multistriata* cells that could be adapted to two additional diatom species (Fig. 5). A description of the protocol step by step follows. With the exception of the number of sonication pulses, the protocol has been applied without any modifications to the other two diatom species.

Reagents and kits:

Artificial sea water (ASW) (Sea salts (Sigma-Aldrich S9883) 3.45%, Sodium bicarbonate 1 mM dissolved in distilled water enriched with 1 × f/2 Guillard medium^42^);
NH_4_F (338,869 Sigma-Aldrich);
NIB (10 mM Tris–HCl pH 7.4; 10 mM NaCl; 3 mM MgCl_2_; 0.1% IGEPAL CA-630);
4.SYBR Green I Nucleic Acid Stain (S7563 Invitrogen);

Instruments:

1. Refrigerated swinging bucket rotor centrifuge with adapters for 50 mL conical tubes;
2. Refrigerated microcentrifuge for 1.5–2 mL tubes;
3. Tip digital sonifier;
4. Fluorescence microscope;
5. Fluorescence-Activated Cell Sorter (FACS).

Procedure

Collection of cells

1. Pellet around 20–30 × 10^6^ cells for 15′ at 1800 xg at 18 °C in 50 mL conical tubes;
2. Collect pelleted cells in 2 mL tubes and spin 10′ at 1500 xg at 18 °C; merge all cells in one 2 mL tube and spin 10′ at 1500 xg at 18 °C;
3. Wash once with 2 mL of filtered ASW: spin 5′ at 1500 xg at 18 °C, cells will collect more easily on the bottom of the tube when they are in ASW; this step is important if enzymatic reactions will follow nuclei isolation as it eliminates potential chelating agents present in natural sea water (i.e.: EDTA) that can inhibit enzymatic activity;

Dissolving diatom cell walls and isolating nuclei:

1. Remove ASW and add 400 μL of NH_4_F solution (10 M, pH 5), mix and incubate 10′ at room temperature vortexing periodically;
2. Add 1.5 mL of filtered ASW to the sample and centrifuge 5′ at 1500 ×g at 18 °C;
3. Remove the supernatant and add 2 mL of filtered ASW, centrifuge 5′ at 1500 ×g at 18 °C;
4. Remove completely the ASW and add 200 μL of ice cold NIB, gently pipette to resuspend the cell pellet and put tubes on ice;
5. Sonicate on ice applying 5 to 10 repetitions of 15′′ pulses (40 W intensity) with intervals of 10′′; intensity and number of pulses need to be adapted to the species;
6. Check cell lysis and nuclei morphology: take a small aliquot (5–10 μL) of sonicated cells, add SYBR Green I (1 × final dilution), put them on a slide to observe under epifluorescent light: if at least 60–70% of cells appears destroyed and free nuclei are visible, stop the sonication procedure, otherwise increase pulse number;
7. Add 1.8 mL of ice cold NIB to the sonicated cells, filter onto 35 μm nylon mesh and add 2 μL of SYBR Green I (10 × final dilution);
8. Dilute the stained sample 10 × with ice cold NIB;
9. Analyze nuclei at the FACS; a combination of Side Scatter and Green Fluorescence (530/40 nm, wavelengths of light that are between 510 and 570 nm) is used to identify and then sort the nuclei population;
10. Sort nuclei using ‘1 drop pure’ sorting criterion and 200 as sorting rate (this setting allows to sort ~ 1,000,000 nuclei in 90′).

## Discussion

The isolation of intact nuclei from cells represents a prerequisite for a number of genome-wide molecular applications that are poorly explored in diatoms, such as Chip-Seq (Chromatin immunoprecipitation and sequencing)^35,42–44^, DNase–seq (Sequencing of DNase I hypersensitive sites)^45^, MNase-Seq (Micrococcal Nuclease digestion with deep Sequencing)^46^, FAIRE–se`q (Formaldehyde-Assisted Isolation of Regulatory Elements with Sequencing) and the already mentioned ATAC-Seq^47^. Nuclei isolation is also needed to generate nuclear proteomes allowing the identification of all the regulatory proteins active in a specific cell state (e.g.: transcription factors, signaling molecules, chromatin associated proteins, etc.)^48,49^. Finally, isolated nuclei are used for Global Run-On sequencing (GRO-seq) assays to measure the rate of transcription for all genes^50^.

Nuclei isolation methods from animal tissues^51^, plants^52^ and yeast^53^ are well established while fewer studies reporting the application of this methodology to phytoplanktonic microorganisms are available so far^54^ and the majority date back to 1976^55^. One paper published in 1966 reports the application to two diatom species (*Ditylum brightwellii* and *Rhizosolenia setigera*) of a nuclei isolation protocol developed for dinoflagellates, but the authors state it was difficult to separate the nuclei from the clutter of broken frustules^56^. To date, a step by step protocol for effective isolation of nuclei from diatom cells is not available.

In this work we present a newly developed method that enabled the extraction of intact nuclei from three different diatom species, the two pennates *P. multistriata* and *P. tricornutum* and the centric *C. diadema*. Our protocol is flexible and allows the isolation of very clean nuclei preparations in shorter times compared to protocols developed for other microalgae, in which separation procedures, such as sucrose gradient centrifugations, are used for the separation of nuclei from cell residues^54,55^.

One of the most important steps for the isolation of intact nuclei from cells is the destruction of the cell wall while leaving intact the nuclear membrane. To accomplish that, it is important to minimize the time of the procedure and the strength of the treatments. Our protocol combines mild chemical treatments with low intensity ultrasound pulses to first weaken and then destroy diatom cell wall allowing nuclei extraction. Chemical treatments involve incubation of cells in acidified NH_4_F solution, that has been previously shown to dissolve the diatom silica and solubilize cell wall proteins such as silaffins^23^. Cells are then resuspended in a low ionic strength NIB that is commonly used to extract nuclei from dissociated animal cells^47^ and nuclei extracted with short pulses of sonication. Ultrasound-based nuclei extraction methods have been previously shown to be highly effective across various organisms and cell types to extract nuclei for chromatin assays without affecting genomic DNA integrity^57^. FAC-sorting is subsequently applied to isolate nuclei and generate pure nuclei preparations and the whole protocol can be performed in less than 3 h. The use of flow cytometry to isolate nuclei for several downstream applications is well documented^58^.

After the isolation of nuclei, it is important to assess their integrity and this can be done using microscopy and checking genomic DNA integrity. Using epifluorescence, confocal and scanning electron microscopy we demonstrated that isolated nuclei appeared clean and their morphology preserved. The observation of *P. multistriata* nuclei at both epifluorescence and confocal microscope revealed in some cases the presence of cell walls fragments surrounding part of the nuclear membrane. Additional pulses of sonication would have removed these cell wall residues, but to preserve nuclei structure we preferred not to add further pulses. Despite the cell wall residues, gDNA could be extracted without treating the extracted nuclei with glass beads (that was instead necessary for extracting gDNA from whole cells); we therefore believe that nuclei may also be accessible to protein extraction as well as enzymatic reactions such as transposition reactions and nuclease digestions, this possibly allowing the application of the above mentioned techniques.

Importantly, *P. multistriata* nuclei observed at SEM before and after the sorting procedure appeared spherical and showed no apparent breakage or leaking of DNA, demonstrating that FAC-sorting is not damaging nuclei. Future work will be aimed at improving the sample preparation procedure for SEM observations to eliminate the precipitates which currently impair the observation of nuclear envelope details such as nuclear pores.

Finally, gDNA extracted from whole cells and isolated nuclei presented comparable profiles when run on Bioanalyzer; despite the method used does not allow to determine the average size of the DNA fragments, the profiles obtained indicate that gDNAs extracted from the isolated nuclei do not present major degradation. These results suggest that for all the tested diatom species, gDNA of good quality could be extracted, further supporting nuclei integrity. DNA integrity and amounts obtained are most likely sufficient for short reads sequencing technologies, while sequencing tests will have to be performed to evaluate suitability for long reads sequencing technologies, where the quantity of input material required could be a limitation. The protocol resulted highly reproducible when applied to *P. multistriata* and *C. diadema* cells. Reproducibility was lower for experiments with *P. tricornutum* cell cultures for which sometimes it was difficult to completely destroy the cells and get nuclei free of cell residues. Modifications in the NH_4_F incubation time and/or in the intensity of the sonication pulses might further improve nuclei isolation efficiency for this species, and similar adjustments will be possibly needed also for other species presenting similar problems.

This protocol has the potential to speed up the application of unexplored techniques in diatoms, potentially advancing the understanding of diatom biology*.* Examples are the epigenetic techniques exploring chromatin accessibility listed above, like the recently established ATAC-Seq that allows the detection of all open sites of the chromatin and the prediction of DNA motifs bound by transcription factors in a specific cell state, approaches that are unexplored in diatoms.

The use of fluorophores that stoichiometrically stain gDNA in the isolated nuclei, coupled to flow cytometry, can also allow genome size assessments^59,60^ representing a valuable alternative to size estimations through post-sequencing genome assembly, that in diatoms has often been problematic because of the difficulties in extracting high molecular weight gDNAs.

The possibility to isolate intact nuclei from diatom cells may also represent the solution to the technical limitations for the application of single cell RNA sequencing (scRNA-Seq) to diatoms. Currently, large diatoms or chain forming diatoms can be problematic for scRNA-seq based on microfluidics since they would clog capillaries; moreover, the presence of the frustule most likely impairs an efficient cell lysis. It is indeed possible to sequence RNA from single nuclei (snRNA-Seq)^40^ even in multiplexed systems^41^. Interestingly, it has been shown that snRNA-seq provides less cell isolation-based transcriptional artifacts and can be applied to frozen specimens^61^. Also, isolated nuclei have been used to generate transcriptome and chromatin accessibility profiles in the same cell^62^. Finally, the application of snDNA-Seq^41^ would represent a powerful approach to understand genomic variation at the level of single individuals in a diatom population.

All the above mentioned applications of the nuclei isolation method make this protocol a potential boost to speed up molecular investigations aimed at shedding light on the complex and still enigmatic mechanisms regulating diatom biology.

## Conclusions

Due to the chemical composition of their cell wall, the extraction of intact organelles from diatoms can be challenging. Combining mild chemical treatments with low intensity ultrasounds and FAC-sorting, and using high resolution microscopy for integrity checks, we developed a protocol to isolate intact nuclei from diatom cells. The developed protocol can be performed in less than 3 h, it has been successfully applied to three diatom species and allowed the extraction of gDNA molecules with low level of fragmentation.

Nuclei isolation from cells is a critical step for several molecular applications such as the generation of nuclear proteomes, epigenetic techniques exploring chromatin accessibility and methods to estimate genome size. Finally, among the most promising applications of the protocol are gDNA and RNA sequencing from single nuclei, bypassing two main problems that are limiting the application of single cell sequencing methods in diatoms: to lyse cells enclosed in glass walls and to use capillaries when working with large and chain forming species.

## Materials and methods

### Algal cultures and sample preparation

*Pseudo-nitzschia multistriata* (MC1334_5), *Phaeodactylum tricornutum* (Pt1 8.6, CCMP2561) and *Chaetoceros diadema* (NA12C1) wild type cells were grown in f/2 Guillard medium^42^ in 12L/12D cycles at 18 °C under white fluorescence neon lamps (Philips TL-D 90) at irradiance of 60 μmol m^−2^ s^−1^. For each experiment 100 mL of cell culture were collected during the exponential growth phase at the following cell densities: *P. multistriata*: 200,000 cells/mL; *P. tricornutum*: 1,600,000 cells/mL, *C. diadema*: 300,000 cells/mL. Cells were collected by centrifugation at 1800 ×g in 50 mL conical tubes using a swinging bucket angle rotor refrigerated centrifuge at 18 °C. Cell pellets were transferred to 2 mL Eppendorf tubes, washed with 2 mL of ASW, centrifuged 10′ at 1500 ×g in a refrigerated microcentrifuge at 18 °C, and incubated for 10′ in 400 µL of NH_4_F (10 M, pH 5) at room temperature.

### Sonication procedure

Before the sonication procedure, NH_4_F treated cells were washed with 2 mL of ASW and then resuspended in 200 µL of cold Nuclei Isolation Buffer (NIB) (10 mM Tris–HCl pH 7.4; 10 mM NaCl; 3 mM MgCl_2_; 0.1% IGEPAL CA-630) in 1.5 mL Eppendorf tubes. Sonication was performed using a Branson 450 digital sonifier (Marshall Scientific) with a 20 kHz 102-C converter (101-135-066R). The 1.5 mL tube was positioned in a small rack on ice and the probe of the sonicator was immersed in the sample at the center of the tube. Depending on the species, 5 to 20 repetitions of 15′′ pulses (40 W intensity) were applied with 10′′ breaks in between.

### Microscopic analyses

For fluorescence and confocal microscopy observations, SYBR Green I (S7563, Invitrogen) (1× final dilution) was used to stain DNA. For all species, intact cells, sonicated cells and FAC-sorted nuclei were imaged using an Axio Imager.Z2 (Zeiss) epifluorescence microscope equipped with an Axiocam 506 mono camera, using a 40× /1.2 W DICIII water immersion objective and the ZEN 2 Blue Edition software. *P. multistriata* and *C. diadema* intact cells and isolated nuclei were imaged with the confocal laser scanning microscope Leica TCS SP8X, using HC PL APO CS2 40× /0.85 dry and 63× /1.2 water immersion objectives (SYBR Green I: excitation at 610 nm, emission filter 495–550 nm; chlorophyll: excitation at 490 nm, emission filter 667–778 nm)*.* For SEM analyses, *P. multistriata* nuclei were fixed for 2 h in 2% glutaraldehyde in NIB and subsequently filtered on a membrane filter of 0.2 µm pore size in a Swinnex filter holder (Millipore, Billerica, Massachusetts, USA); nuclei were then washed with 20 mL of NIB and dehydrated with a graded ethanol concentration (30%, 50%, 70%, 90%, 100%) followed by two washes in 100% ethanol; finally, samples were critical point dried, gold sputter coated and observed with a JEOL JSM 6700F scanning electron microscope (JEOL Ltd, Tokyo, Japan) with a 5 kV accelerating voltage.

The final images were prepared using Adobe Photoshop CS6 software v 21.2.4 and Adobe Illustrator CC 2019 v 23.0.1.

### Nuclei FAC-sorting procedure

Before sorting, 1.8 mL of ice cold NIB was added to 200 µL of sonicated cells and the entire volume was passed through a 35 µm nylon mesh cell strainer (Falcon) to remove aggregates. Cell suspensions were stained with SYBR Green I (10× final dilution). Nuclei sorting was performed with a Becton Dickinson Influx cell sorter (BD Biosciences, Franklin Lake, USA) equipped with a 488 nm argon laser and 100 µm nozzle orifice, operating at 27 psi pressure and 39.22 kHz, with a sort rate of 200 events per second. The combination of Side-Scatter (SSC) and Green Fluorescence (530/40 nm) was used to discriminate fluorescent nuclei population from debris. The ‘1 drop pure’ sorting mode was used for maximal sort purity. The accuracy of nuclei sorting was verified by fluorescence microscopy observations. Nuclei were sorted in a collection tube containing 50 μL of ice cold NIB. Data acquisition and recording were achieved with the BD FAC Software; graphs were drawn with FCS Express 6 Flow v 6.06.0025, DeNovo Software, USA.

### Genomic DNA extraction and quality control

Sorted nuclei and whole cells were collected by centrifugation (10′, 1500 ×g) in a refrigerated microcentrifuge at 18 °C. Genomic DNA extractions were performed using the DNeasy Blood & Tissue Kit (Qiagen, Cat No./ID: 69504) following manufacturer’s instructions with the following modifications: cell pellets were resuspended in 180 μL of Qiagen ATL buffer and then incubated with glass beads (0.5 mg of acid washed glass beads, Sigma Aldrich) on a Vibrax at maximum speed at 56 °C for 20′ (nuclei were not subjected to incubation with glass beads); 30 μL of Qiagen AE buffer were used to elute both cell and nuclei extracted gDNAs (see also https://dx.doi.org/10.17504/protocols.io.bdwxi7fn). gDNA yields were quantified using a Qubit dsDNA HS Assay Kit and the Qubit Fluorometer (Thermo Fisher) following manufacturer’s specifications. 1.5 ng of each gDNA were loaded on Agilent High Sensitivity DNA Chips (Agilent Technologies) and run on an Agilent 2100 Bioanalyzer System following manufacturer’s instructions.

This protocol is also available at 10.17504/protocols.io.bmj5k4q6.

## Supplementary Information

Supplementary Information 1.
