# Supplementary material for: An optimised method for intact nuclei isolation from diatoms
Source: Sci Rep. 2021 Jan 18;11:1681. doi: 10.1038/s41598-021-81238-z (PMC7813820; doi:10.1038/s41598-021-81238-z)
Supplement: Supplementary file 1 — Supplementary Information 1. [file 41598_2021_81238_MOESM1_ESM.pdf]

# An optimised method for intact nuclei isolation from diatoms

Rossella Annunziata[1,*], Cecilia Balestra[1], Pina Marotta[1], Antonella Ruggiero[1], Francesco Manfellotto[1], Giovanna Benvenuto[1], Elio Biffali[1], Maria Immacolata Ferrante[1,*]

[1] Stazione Zoologica Anton Dohrn, 80121 Napoli, Italy.

## Supplementary Figure 1

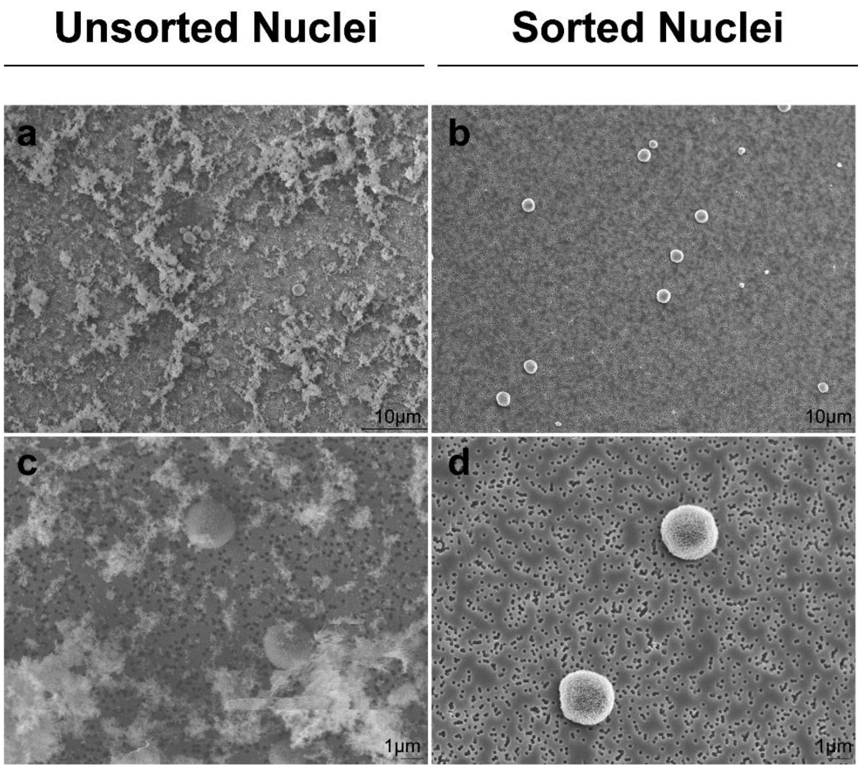


**Supplementary Figure 1. SEM analysis of *P. multistriata* nuclei before and after FAC-sorting.** (a-c) *P. multistriata* nuclei before FAC-sorting at two different magnifications; cell debris is clearly visible. (b-d) *P. multistriata* nuclei subjected to FAC-sorting after cell sonication; nuclei morphology appears similar to unsorted nuclei but cell debris is absent.

## Supplementary Table 1

| $NH_4F$ solution incubation time | Glass beads Treatment | Result |
|---|---|---|
| None | 15’ - 30’ | Mostly intact cells – no nuclei |
| 3’ | 15’ - 30’ | Mostly intact cells – no nuclei |
| 5’ | 15’ - 30’ | Mostly intact cells – no nuclei |
| 10’ | 15’ | Few broken cells – no nuclei |
| 10’ | 20’ | 30% broken cells – few nuclei |
| 10’ | 30’ | 50% broken cells – few nuclei |

**Supplementary Table 1.** Different combinations of incubation times in $NH_4F$ solution and glass beads treatments tested to break *P. multistriata* cells and isolate intact nuclei.

## Supplementary Table 2

| $NH_4F$ solution incubation time | Sonication intensities (W: Watt) | Number of Pulses; Duration of pulses | Result |
|---|---|---|---|
| 3’ - 5’ | 20 W | 1; 30” - 3’ | Mostly intact cells – no nuclei |
| 3’ - 5’ | 40 W | 1; 30” - 3’ | Mostly intact cells – no nuclei |
| 10’ | 20 W | 1; 30” - 3’ | 50% broken cells after 3’ – many nuclei |
| 10’ | 40 W | 1; 30” - 2’ | 60 - 70% broken cells after 2’ – many nuclei |
| 10’ | 40 W | 3 - 11; 15” | 60 - 70% of broken cells were obtained applying from 3 to 11 pulses depending on the strain – good intact nuclei recovery |

**Supplementary Table 2.** Different combinations of incubation times in $NH_4F$ solution and sonication pulses tested to break *P. multistriata* cells and isolate intact nuclei.
